# 3D Printing in Prosthetics, Orthotics and Assistive Technology: Myth and Reality

**DOI:** 10.33137/cpoj.v6i2.42222

**Published:** 2023-12-22

**Authors:** S.U Raschke

**Affiliations:** British Columbia Institute of Technology, Applied Research MAKE+ (retired), 3700 Willingdon Avenue, Burnaby, Canada.

**Keywords:** Prosthetics, Orthotics, Additive Manufacturing, 3D Printing, Digital Production Chain, Design, Rehabilitation

## Abstract

3D printing initially captured the public eye when mainstream media began writing about Enabling the Future, a volunteer network that had begun designing and 3D printing prosthetic hands. Many of the stories focused on how this technology was going to disrupt the prosthetic sector. The response from prosthetists was skepticism and concern, in particular warning that 3D printed components would not be robust enough to withstand the activities of daily living. Moreover, they emphasized that fit problems could potentially cause more harm than good. Several years on, this issue explores currant usage and experiences with the technology in prosthetics and, to a limited extent, orthotics.

## INTRODUCTION

The BCIT MAKE+ Department, of which I was a part, has over two decades of experience in 3D printing in rehabilitation and health application, starting in 2001, with a Canadian Foundation for Innovation (CFI) grant to establish the Centre for Rehabilitation Engineering that Enables (CREATE) of which I was the Principle Investigator and in collaboration with Dr. Gary Birch and the Neil Squire Society, a not-for-profit that helped developed unique assistive technology for persons with high level spinal cord injury and continues to do so today through the Makers Making Change program.^1^ The focal point of the CREATE grant was a Stratysis 3D printer to be used for prototyping assistive technology and biomedical devices.^2^

Despite early fears expressed on the part of prosthetic and orthotic clinicians, our team was optimistic that this technology was a fit for the sector. Our work with Fused Deposition Modelling (FDM) printing in the biomedical and rehabilitation setting, with that initial 3D printer, had already given us some idea of the benefits and the limitations of the technology. Our optimism was reciprocated by both commercial clients seeking our applied research experience, as well as by funding agencies. Over the past 22 years we carried out a range of projects that 1) tested prosthetic sockets, including 3D printed models 2) examined prosthetic and orthotic digital production chains, either with commercial clients and as a graduate student project and 3) continued to use our 3D printers to prototype designs across a range of rehabilitation and biomedical applications. In 2011 our team had expanded with the awarding of a Canada Research Chair, to Dr. Jaimie Borisoff. a former post-Doc of Dr Gary Birch, whose lab provided 2 further FDM printers, plus a wider range of production tools (laser cutter, water jet cutter, etc.), Funding was also included for post-Doc positions and student projects. This was housed under the Rehabilitation Engineering (RED) Lab, into which CREATE was integrated.

It is this background from which I write this editorial today, which unlike my typical editorials, is a more personal one. It is based on an introduction I gave to a workshop on 3D Printing Assistive Technology, Orthotics and Prosthetics^3^ recently hosted at BCIT as part of our latest initiative: the BCIT Centre for Applied Research and Innovation's Advanced Additive Manufacturing Hub (AAMTECH).^4^ Funded by the Teck Copper + Health Initiative and PacifiCan, the Hub builds on our two decades of experience to further support practical research on the uses of Additive Manufacturing (AM) for Health Applications.

At the workshop, I spoke about my early research career as an orthotist in the late 1980s participating on projects at UBC's Medical Engineering Resource Unit (MERU) led by Carl Saunders before his spinning the technology of the university setting as Vorum Research. In one conversation with Carl, I remember saying that what the field of prosthetics and orthotics needs is objective CAD/CAM systems which could create data informed designs. He said that what I was asking for was not possible due to the lack of data and understanding of the structural and mechanical properties of prosthetic and orthotic devices. While this may not have been possible at that time, his answer helped lead to my decision to pursue graduate studies and a research career. That decision was also driven by my repeated questions to my clinical supervisors of: “Why make the orthosis or prosthesis this way?”. Questions that were never answered with objective, evidence supported answers.

Much changed in the subsequent four decades. There was the development and expansion of evidence-based measures in the clinical setting in particular for lower limb prosthetics^5^ which set the stage for objective consideration of prosthetic components design. At the same time the patents on 3D printers began expiring, expanding availability and lowering costs making 3D printers available in the home and classroom. Printers that were increasingly sophisticated. At the industrial level access to additive manufacturing technology such as metal printers and carbon fiber printers also began to improve. This evolving landscape allowed new communities of practice to emerge, who had identified gaps in the prosthetic and orthotic provision process that they believed they could bridge.

Some of the speakers at the aforementioned workshop have contributed to this Special Edition. Other authors write about their experiences with practical and research experience in 3D printing in prosthetic and orthotic devices. They share, in their words, how far we have come both from the initial use of CAD/CAM and 3D printing in orthotics and prosthetics and its more recent explosion into the public eye a decade ago, providing a pragmatic perspective of where we are now. Research into further developing additive manufacturing for this sector continues alongside active exploration of how what is developed can provide value to both patient and clinicians in the clinical setting. This is supported by ongoing research generating data on materials, biomechanical and structural engineering data from device testing projects, clinical outcome measure application research and, as it becomes more accessible, data from an amputee registry development initiative, being led by Dr Kenton Kaufmann at the Mayo Clinic.^6^ The synergies created by the crossover between clinical and engineering research will support the development of expert models that I had been looking for almost four decades ago.

## CONCLUSION

Authors in this issue share opportunities and obstacles faced as they engaged with 3D printing and the digitization of the sector. Their experiences include the important message that 3D printers themselves do not do research or provide clinical care. They are just another tool that add capacity and capabilities, but they must be paired with skilled persons engaged in the prosthetic or orthotic design and provision process. A 3D printer on its own is of little use. What is also becoming clear is that benefits cannot be harnessed or obstacles overcome without reaching out across skills sets to work in interdisciplinary teams. It is incumbent on prosthetists and orthotists to reach out and collaborate with researchers and technology developers. This ensures that the digital production chains and innovative additively manufactured designs being developed do not leave them behind, potentially resulting in less-than-optimal service for their patient-clients.

## CALL TO ACTION

In closing, I encourage the clinical readers of this Special Edition to being open to digital production chains and additive manufacturing processes and to actively reach out to researchers and technology developers with offers to collaborate and contribute, so that the systems being developed serve them as opposed to, in future, becoming their masters. For readers in the researcher and developer communities, I urge you to make a commitment to including prosthetists and orthotists in all stages of research to draw on their empirical knowledge in order to ensure the outcomes are comprehensive and robust. This requires both groups to go look beyond their comfort zones and learn from each others, ultimately benefiting the individuals they both groups aim to serve, those persons requiring assistive technology to maintain an active and fulfilling quality of life.

## DECLARATION OF CONFLICTING INTERESTS

I have no conflicts to interest to declare.

## SOURCES OF SUPPORT

None.

## AUTHOR SCIENTIFIC BIOGRAPHY

**Figure:**
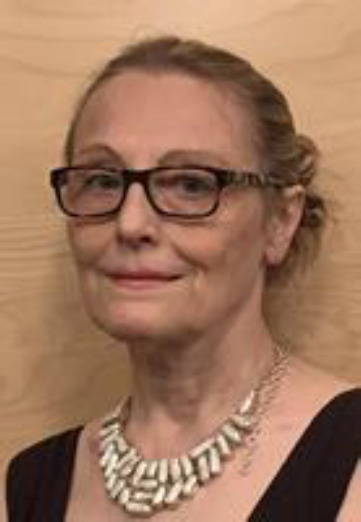


**Dr Silvia Raschke**, PhD, is an applied researcher (retired) with the British Columbia Institute of Technology MAKE+ group. She specializes in evaluation and product development projects in rehabilitation engineering with a focus on prosthetics and orthotics. In 2013 she and collaborator, Dr. Michael Orendurff, PhD won the Thranhardt Prize for their paper: “Can You Tell Which Foot is Which?”, the first double blind prosthetic foot evaluation that included community ambulation. She is currently involved in a diverse range of projects, including orthotic aspects of exoskeleton design, curriculum development and acting as a mentor to a team of young researchers who are doing a project examining Glass Ceilings in Prosthetics and Orthotics. She is Editor-in-Chief of the Canadian Prosthetics and Orthotics Journal and Chair of the US Veterans Affairs Rehabilitation Research and Development (RR&D) Subcommittee on Rehabilitation Engineering and Prosthetics/Orthotics.
